# The Treatment of Acute Rheumatism and Its Complications

**Published:** 1893-03-18

**Authors:** 


### BRISTOL CHILDREN'S HOSPITAL.

### The Treatment of Acute Rheumatism and Its Complications.

The members of the staff of this hospital see their own out-patients, and consequently select the cases for admission as in-patients. Whatever may be the drawback to this system, it has one decided advantage, it enables the physician or surgeon under whose charge a case may be to keep it under his own eye from first to last. The child is examined, as far as may be considered necessary, in the out-patients' department, and then, if considered suitable for admission, is sent over to the hospital in charge of the nurse who is generally present whilst out-patients are being seen. On admission, the little patient's temperature is at once taken; if this is not over 100 degrees Fahr. the child is bathed, if higher, a careful sponging with warm water in bed is substituted, great care, of course, being taken as to drying. Should pain in the joints be complained of, or should any swelling be apparent, each joint is carefully wrapped in cotton-wool and lightly bandaged, and the chest covered with a jacket made from Gamgee's tissue, it being generally considered advisable that the child should sleep in blankets. On the physician's visit to the wards the patient is thoroughly examined, the heart's condition, &c., made out, and the necessary treatment and diet ordered. Should the case be a mild one, without heart symptoms being then present, some mild saline, such as citrate of potash, or the liquor ammon. acet. is ordered, whilst the child's being kept at complete rest in bed places the heart in the most favourable circumstances by doing as little work as possible to meet the possibly coming storm, for it is well to bear in mind that the mildness of an attack as regards temperature and joint symptoms in its early stage is no criterion by which to judge of the probability of the occurrence of cardiac complications; indeed, it is remarkable how endocarditis will develop in cases of rheumatism in children with little or no joint pain or swelling, and it is well to frequently examine the condition of the heart, bearing this fact in mind. The percentage, too, of cases in which endocarditis or peri-endocarditis occurs in children is much larger than in adults, and may be taken to be about 80 per cent. of the whole.

Should the case be a severe one, with many of the joints affected, and much fever present, salicylate of soda is usually prescribed in doses varying according to the age of the child—say, from three to ten grains every four hours for children from five to ten years old, for three or four days, and then only three times a day.

The diet in both mild and severe cases is milk, given with or without soda or potash water, according to circumstances, the alkaline waters being usually ordered when there is sickness or a tendency for the milk to disagree. This diet is strictly adhered to as long as there is any fever, the alterations in diet being very gentle and gradual, as a common cause of relapse is allowing beef-tea, soup, or meat too early in convalescence. Before ordering these rice or custard is provided, and is allowed if the temperature has been normal for a week and all pain absent for several days.

Of the complications in acute rheumatism the most usual are peri and endo carditis (the latter the commoner of the two), or both together, and a disease which, whilst generally preceding or following on a rheumatic attack, is sometimes found present along with acute rheumatic symptoms, viz., chorea. The experience of the physicians at the Bristol Children's Hospital seems to be strongly in support of the accepted theory that acute rheumatism is hereditary. Frequently a child is presented to one or other of them as ailing with no other symptom, perhaps, than a little sore throat, who, upon auscultation, is found to have a mitral systolic bruit, and whose parents, on being carefully questioned, admit to having had rheumatic fever possibly more than once, and a like condition can sometimes be traced in a generation further back. A remarkable case, illustrating this tendency, was published in Vol. XXV. of Guy's Hospital Reports, "where, with a rheumatic strain in both father and mother, five out of a family of six children under fifteen, all but a baby of fourteen months, had either had rheumatism or heart disease."

The treatment of endocarditis at the Bristol Children's Hospital varies somewhat according to the partiality of the different physicians for certain remedies, but there seems to be a pretty general use of salicylate of soda, even after cardiac symptoms have developed. This drug is supposed by some authorities to be of little service after pain has ceased and heart symptoms are prominent, and is justly dreaded on account of its depressing effects; but given as in this hospital, in combination with carbonate of ammonia, and its effect carefully watched, its continuance for a while will be found very beneficial.

If pericarditis (or endo-pericarditis) ensues, the treatment is mainly directed toward quieting excessive cardiac action, and giving the heart rest. A poultice of linseed meal, spongio piline, or a small blister (latter rarely) is applied over the precordium, at the same time opium, usually in the form of Dover's powder, is administered; two or three grains, three or four times in twenty-four hours, being considered sufficient for a child of eight years old. If the heart's action is very weak and irregular, digitalis in the form of the tincture two to five minims three times a day is exhibited, which aids to rest the heart by prolonging the pause, or sometimes belladonna is prescribed instead.

In cases where, in spite of all the above care and attention, the heart continues to fail and the pulsations increase in rapidity, accompanied by dulness at both bases and rapid breathing, brandy is freely administered, and sometimes seems to control the troublesome vomiting, the occurrence of which is so serious at this stage of the disease. All these cases are kept strictly in bed for several weeks after all troublesome symptoms have passed away, it being recognised that great care is necessary for a long time to prevent anything approaching cardiac excitement.

The last complication we shall be able to refer to at present is chorea. The treatment of this complaint amongst the out-patients does not seem to be so satisfactory as could be wished, and undoubtedly the cause of this is the difficulty of giving the little patients complete rest. As in-patients, however, the case is very different, a few days' rest in bed and most of them begin to improve, some in a marked manner. The treatment usually begins with a full dose of castor oil, or some other suitable aperient, to well clear out the bowels; sleeplessness, if present, is counteracted by a dose of Dover's powder, and when the child is comfortably settled down, arsenic (either alone or with iron) is administered; Liq. Arsenicalis, from three to ten minims in half an ounce of water, is given three times a day after food, according to the age of the child, the dose being gradually increased if necessary. Should this treatment fail to satisfy, sulphate of zinc, three to ten grains three times a day, is given, but as a rule the arsenic does its work well.

With regard to diet in chorea, should the temperature be above normal, a full milk diet is ordered, and persevered with until the temperature falls, otherwise the fare known as "ordinary" hospital diet is preferred.

